# Early hematoma retraction in intracerebral hemorrhage is uncommon and does not predict outcome

**DOI:** 10.1371/journal.pone.0205436

**Published:** 2018-10-09

**Authors:** Ana C. Klahr, Mahesh Kate, Jayme Kosior, Brian Buck, Ashfaq Shuaib, Derek Emery, Kenneth Butcher

**Affiliations:** 1 Division of Neurology, Department of Medicine, University of Alberta, Edmonton, Canada; 2 Department of Neurology, Christian Medical College Ludhiana, Ludhiāna, India; 3 Department of Radiology, University of Alberta, Edmonton, Canada; University of L’Aquila, ITALY

## Abstract

**Background:**

Clot retraction in intracerebral hemorrhage (ICH) has been described and postulated to be related to effective hemostasis and perihematoma edema (PHE) formation. The incidence and quantitative extent of hematoma retraction (HR) is unknown. Our aim was to determine the incidence of HR between baseline and time of admission. We also tested the hypothesis that patients with HR had higher PHE volume and good prognosis.

**Methods:**

This was a retrospective single-centre study in which serial planimetric volume measurements of the total hematoma volume (parenchymal (IPH) and intraventricular (IVH)) and PHE were performed in ICH patients with baseline non-contrast computed tomography (CT) completed within 6 hours of onset and follow-up CT 24 (±12) hours from symptom onset. HR was defined as a decrease in volume of >3ml or >15%, and hematoma expansion (HE) as an increase of >6ml or >30%. All other patients were categorized as stable hematoma (HS). Good outcome was defined as modified Rankin Scale (mRS) 0–2 at 90 days.

**Results:**

A total of 136 patients (mean age = 69.3±13.39 years, 58.1% male) were included. Median (interquartile range) baseline total hematoma volume was 14.96 (7.80, 31.88) ml. HR >3ml and >15% occurred in 6 (4.4%) and 8 (5.9%) patients, respectively. Neither definition of HR was associated with follow-up PHE (p>0.297) or good outcome (p>0.249). IVH was the only independent predictor of HR (p<0.0241).

**Conclusions:**

Early HR is rare and associated with IVH, but not with PHE or clinical outcome. There was no relationship between HR, PHE, and patient prognosis. Therefore, HR is unlikely to be a useful endpoint in clinical ICH studies.

## Introduction

Hematoma size is one of the main predictors of disability and mortality in intracerebral hemorrhage (ICH).[1] Dynamic changes occur within the first 6 hours, including hematoma expansion (HE) in up to one third of ICH patients.[2–5] HE is associated with worse prognosis.[1,4]

The coagulation process, which starts within minutes of ICH onset and involves the polymerization of fibrin into a clot, stabilizes the hematoma.[1] Hemostasis is associated with perihematoma edema (PHE) formation, possibly related to serum separation from the hematoma.[6,7] Early clot resolution may be a predictor of better patient outcome.[8]

In this study, we aimed to determine the incidence of early hematoma retraction (HR) in ICH. We hypothesized that PHE is larger in those patients with HR. We also tested the hypothesis that HR is associated with better prognosis.

## Methods

### Patients

This was a retrospective single-center study of patients presenting with primary ICH within 24 hours of onset. Patients presenting to our hospital between January 2007 and March 2017 were included. All patients were included in prospective studies of ICH conducted at our center and provided informed written consent to have data from their medical records used in research.[9–12] These studies were observational or randomized interventions of blood pressure or hemostatic treatment, neither of which affect hematoma size.[10,13–15] The protocols were approved by the human research ethics board at the University of Alberta.

Exclusion criteria included patients <18 years, diagnostic CT performed later than 6 hours and follow-up CT later than36 hours from symptom onset, evidence of secondary ICH, surgical intervention, including extraventricular drainage, and vasculitis. Patients with anticoagulant-associated ICH (AAICH) were included if they were treated with Prothrombin Complex Concentrate within 1 hour of the baseline CT scan.

### Clinical assessments

Baseline demographic information as well as medical history were obtained from clinical records. National Institutes of Health Stroke Scale (NIHSS) score, which reflects the level of impairment due to the stroke, was obtained at time of admission. The lowest NIHSS score is 0, indicating no neurological deficits, and the highest is 42.[16] Functional outcome was assessed with the modified Rankin Scale (mRS) 90 days post-ictus. The mRS measures the degree of disability from 0 (no symptoms) to 6 (deceased).[17]

### Imaging protocol and analysis

Baseline and follow-up CT scans consisted of 4.8-mm sections (120 KV[peak], 200 mA per section) through the entire brain (27–33 sections with a 512×512 matrix). Planimetric assessment of hematoma volumes was performed using Quantomo software.[18] A semi-automated threshold-based seed growing algorithm was used to define hematoma boundaries. Intraventricular and intraparenchymal segmentation was completed manually. Segmentation and volume measurements were completed by two investigators (AK and KB) using consensus, and repeated on two different occasions, without reference to the previous measurements. Peri-hematoma edema (PHE) volumes were measured using Analyze 11.0 (Biomedical Imaging Resource)[19]. The visibly hypodense region was manually outlined and a threshold of 5 to 23 HU was used to objectively define perihematoma volumes, as previously described.[20] Relative edema was calculated as the ratio of this PHE volume to hematoma volume.[21]

### Outcomes

The primary outcome was HR, defined as an absolute volume decrease (including intraparenchymal (IPH) and intraventricular (IVH) components) between the baseline and follow-up (24 hour) scans of >3ml. A relative volume decrease of >15% between baseline and follow-up scans was a secondary outcome. The definition of HR was chosen arbitrarily. Hematoma expansion (HE) was defined as >6ml and >30% increase in volume between scans, based on previous studies [4,5]. All other patients were defined as having a stable hematoma volume (HS). Other secondary outcomes included IPH and IVH volume change, as well as PHE volume change between baseline and follow-up scans and the proportion of patients with a good clinical outcome, defined as mRS 0–2 at day 90.

### Statistical analysis

Statistical analysis was performed using SPSS Statistics 21.0 2008 (IBM, Armonk, New York). Baseline characteristics were compared using independent *t* tests or Mann–Whitney U tests. Pearson χ2 or Fisher exact tests were used to compare frequencies of categorical outcomes. Multinomial logistic regression was used to determine the predictive value of independent variables on HR and HE (HS was the reference group). Linear regression was used to determine the relationship between PHE and HR. The intra-class correlation coefficient (ICC) was calculated to assess reliability of the consensus ICH volume measurements performed by the raters on different occasions.

## Results

Of the 332 patients presenting with primary ICH, 136 were included in the study (mean age ± SD = 69.31 ± 13.39, 58.1% male; see all raw data S1 File). Baseline scans were obtained 1.96±1.30 hours from onset and follow-up imaging was completed 21.18±6.18 hours later. Median (IQR) baseline total hematoma volume was 14.96 (7.80, 31.88) and NIHSS was 12 (8, 17.50). The ICC for hematoma volumes was 0.996 (95% CI [0.994, 0.997], *p* = 0.0001). The reasons for exclusion were secondary ICH (N = 43), baseline CT > 6 hours and/or follow-up (24 hour) CT not completed within 12–36 hours (N = 134), vasculitis (N = 2), extraventricular drain (N = 13), and surgical decompression (N = 4).

### Absolute hematoma volume change

**Fig 1:**
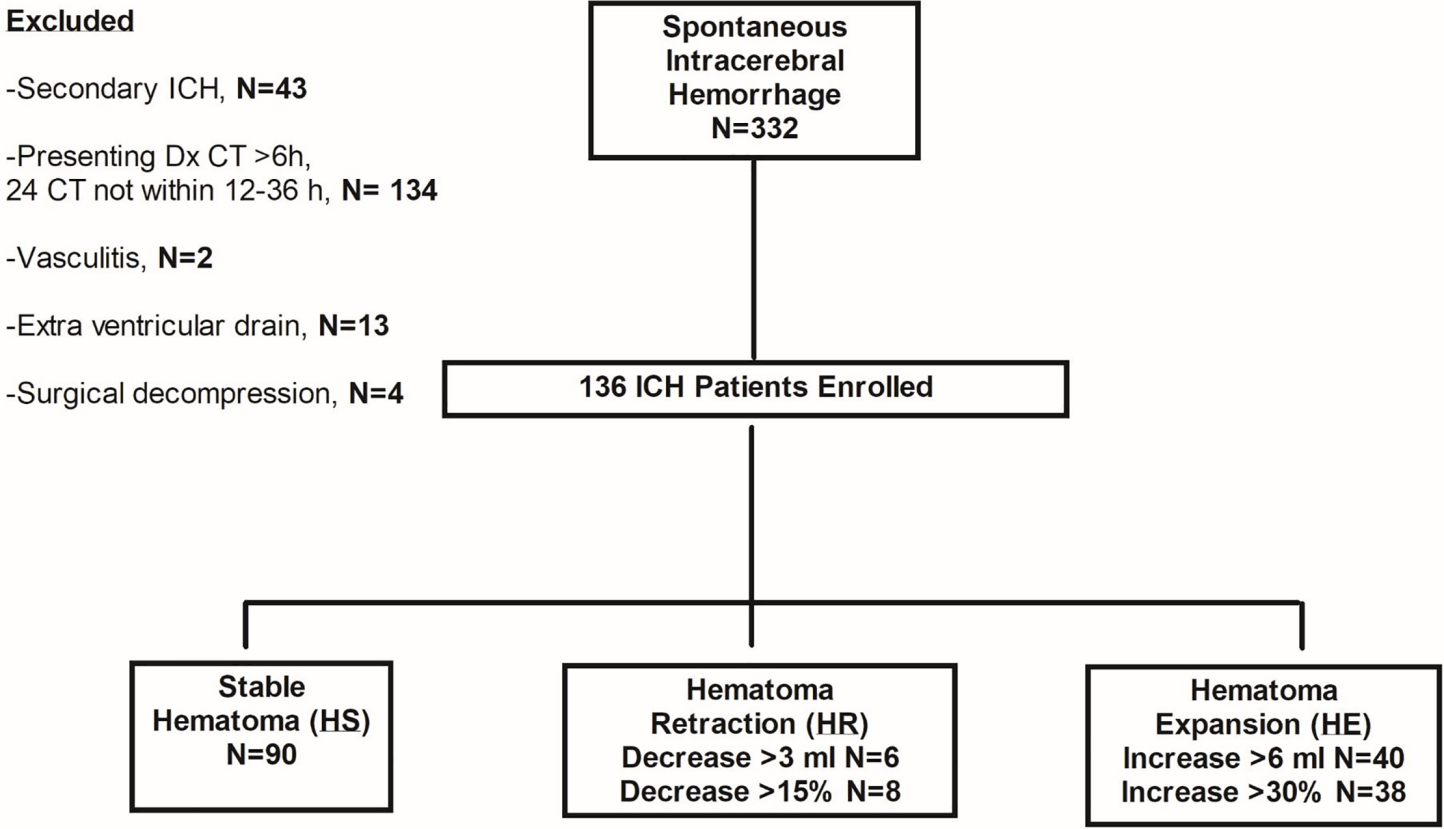
Study profile.

**Fig 2:**
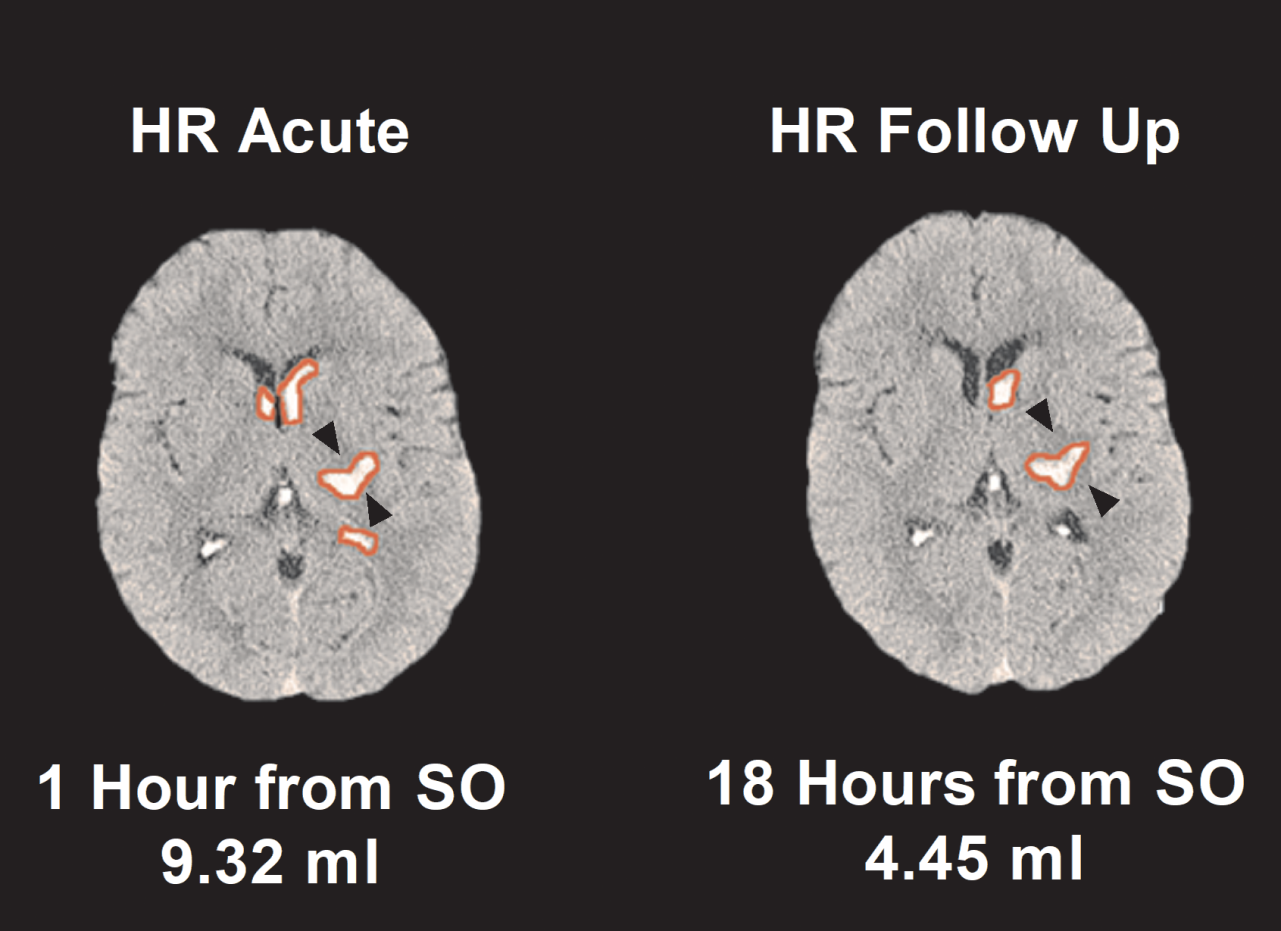
Example of HR in a patient with a left thalamic ICH and associated IVH. Although the total hematoma volume decreased by 4.87 ml or 52.25%, the IPH component was stable. The apparent decrease was associated with a loss of measurable IVH (4.02 ml or 81.05%). The acute perihematomal edema (PHE) was 0.5 ml and the follow-up edema was 1 ml (indicated by the black arrows).

Most patients (90 (66.2%)) had stable (HS) volumes (Fig 1). Hematoma expansion (HE) of >6 ml was seen in 40 (29.4%) of patients (Table 1). Patients with HE were more likely to be AAICH (Table 2). Six (4.4%) patients had objective evidence of HR of >3 ml. All HR patients had an IVH on the baseline CT scan (Fig 2). Two HR patients had an AAICH, which was corrected with PCC and vitamin K. Both patients had intraventricular extension. In the 6 HR patients, the median (IQR) difference in total volumes was 4.50 (3.60, 4.80) ml. Most of this apparent decrease in volume between the two scans was due to a change in the IVH measurement (3.11 (2.1, 5)) ml, rather than IPH (1.18 (-0.30, 2.60) ml) when compared to HS (*p* = 0.0001). The only independent variable associated with HR was IVH (Table 3). Predictors of HE included acute ICH volume (OR = 1.059, 95% CI [1.032, 1.087], *p* = 0.0001) and AAICH (OR = 6.609, 95% CI [1.605, 27.208], *p* = 0.006).

**Table 1:** Imaging characteristics (groups defined using absolute ICH volume change).

	Stable Hematoma	Hematoma Retraction	Hematoma Expansion	P value
	HS; N = 90	HR, N = 6	HE, N = 40	
**Symptom onset to acute CT (Hours); Median (IQR)**	2 (1, 3)	2.5 (1, 3)	1 (1, 2)	0.121
**Acute CT to Follow-up CT (Hours); Median (IQR)**	22 (18, 25)	18.5 (13, 22)	20.5 (14, 26.5)	0.363
**Location**				**0.001**
Deep	63	5	22	
Lobar	15	0	18	
Brainstem	7	0	0	
Cerebellum	5	1	1	
**Acute IPH volume (ml); Median (IQR)**	8.67 (5.07, 15.48)	7.03 (6.50, 27)	31.44 (20.88, 55.91)^a^	**0.0001**
**IVH**	28 (31%)	6 (100%)^b^	13 (32%)	**0.0001**
**Acute IVH volume (ml); Median (IQR)**	0 (0, 1.62)	4.73 (2.6, 5.7)^b^	0 (0, 2.65)	**0.006**
**Acute total ICH volume (ml); Median (IQR)**	9.58 (5.10, 19.68)	14.33 (11, 29.6)	31.88 (22.05, 57.69)^a^	**0.0001**
**Follow up IPH volume (ml); Median (IQR)**	9.35 (5.01, 18)	8.26 (5, 24.4)	56.47 (37.53, 87.72)^c^	**0001**
**Follow-up IVH(ml); Median (IQR)**	0 (0, 2.40)	0.94 (0.4, 2.40)	1.63 (0, 7.94)^a^	**0.004**
**Follow-up total ICH volume (ml); Median (IQR)**	11.20 (5.80, 22.84)	10.21 (7.40, 24.80)	58.62 (40.25, 98.33)^c^	**0.0001**
**Follow-up PHE (5–23 HU, ml); Median (IQR)**	2 (1.2, 3.9)	1.7 (0.5, 3.3)	7.8 (4.7, 13.4)^c^	**0.0001**
**Follow-up relative PHE (5–23 HU); Median ± IQR**	0.25 (0.11, 0.52)	0.21 (0.06, 0.32)	0.14 (0.09, 0.27)^a^	**0.027**

^a^vs. HS *p*<0.001

^b^vs. HE and HS *p*<0.006

^c^vs. HS and HR *p*<0.030

**Table 2:** Baseline clinical characteristics and outcomes (groups defined using absolute volume change criteria).

	Stable Hematoma	Hematoma Retraction	Hematoma Expansion	P value
	HS, N = 90	HR, N = 6	HE, N = 40	
*Baseline*				
**Age; Mean ± SD (years)**	68 ± 13	68 ± 16	72 ± 14	0.165
**Male**	52 (58%)	5 (83%)	22 (55%)	0.382
**NIHSS, Median (IQR)**	10 (8, 15)	11 (9, 15)	15 (10, 20)^a^	**0.021**
**Baseline Systolic BP (mmHg); Median (IQR)**	181.8 (161, 202)	174.3 (152, 191)	173 (156, 194)	0.474
**Baseline Diastolic BP (mmHg); Median (IQR)**	90.50 (81, 109)	94.5 (87, 119)	88 (75, 102)	0.294
**Past History of Stroke**	20 (22%)	2 (33%)	4 (10%)	0.261
**Anticoagulant-associated ICH**	4 (4.8%)^b^	2 (40%)	9 (25.7%)	**0.002**
**Diabetes**	26 (30%)	2 (33%)	8 (21%)	0.537
**Glucose (mmol/l), Median (IQR)**	7.30 (5.7, 8.5)	7.20 (6.5, 8)	7.20 (5.80, 8.30)	0.842
**Hypertension**	67 (75%)	5 (83%)	29 (74%)	0.893
**Aspirin**	20 (24%)	2 (40%)	7 (21%)	0.597
*Outcome*				
**90 Day mRS**	3 (2, 4)	3 (3, 4)	6 (4, 6)^c^	**0.0001**
**mRS 0–2**	35 (56.5%)	3 (60%)	3 (11.1%)^c^	**0.0001**

^a^vs. HS *p*<0.0292

^b^vs. HE and HR *p*< 0.0356

^c^vs. HE and HS *p*<0.0342

**Table 3:** Predictors of hematoma retraction.

Factors	OR [95% CI]	P value
***HR >3 ml***		
Acute ICH volume	0.99 [0.941, 1.061]	0.980
Symptom onset to acute CT	0.93 [0.807, 1.071]	0.310
Symptom onset to 24 hour CT	1.09 [0.578, 2.064]	0.785
**IVH**	**28.51 [1.55, 523.540]**	**0.0241**
***HR > 15%***		
Acute ICH volume	0.92 [0.849, 1.003]	0.059
Symptom onset to acute CT	0.82 [0.432, 1.550]	0.538
Symptom onset to follow-up CT	1.06 [0.917, 1.230]	0.425
**IVH**	**37.955 [3.642, 395.600]**	**0.002**

### Relative hematoma volume change

When defined using relative volume changes, most patients (90 (66.2%)) had HS. Hematoma expansion (HE) of >30% was seen in 38 (27.8%) of patients, who were also more likely to be AAICH (Table 4). Eight (5.9%) patients had HR >15%, 7 of whom had intraventricular extension. The only independent predictor of relative HR was IVH (Table 3).

**Table 4:** Imaging characteristics (groups defined using relative ICH volume change).

	Stable Hematoma	Hematoma Retraction	Hematoma Expansion	P value
	HS; N = 90	HR, N = 8	HE, N = 38	
**Symptom onset to acute CT (Hours); Median (IQR)**	2 (1, 3)	1.5 (1, 3)	1 (1, 2)	0.226
**Acute CT to Follow-up CT (Hours); Median (IQR)**	22 (18, 25)	22 (18.5, 28.5)	23 (14, 26)	0.363
**Location**				0.521
Deep	57	7	26	
Lobar	25	0	8	
Brainstem	5	0	2	
Cerebellum	3	1	2	
**Acute IPH volume (ml); Median (IQR)**	10.55 (5.93, 30.13)	7.03 (6.25, 8.33)	18.95 (8.95, 30.50)	0.070
**IVH**	29 (29.01%)	7 (87.5%)^a^	11 (28.9%)	**0.005**
**Acute IVH volume (ml); Median (IQR)**	0 (0, 2.5)	4.73 (2.2, 7.30)^b^	0 (0, 0)	**0.002**
**Acute total ICH volume (ml); Median (IQR)**	13.76 (6.80, 32.69)	11.78 (8.61, 16.90)	20.37 (10.00, 31.98)	0.504
**Follow up IPH volume (ml); Median (IQR)**	10.06 (6.04, 30.80)	7.06 (5.20, 8.67)	38.39 (14.52, 70.10)^c^	**0.0001**
**Follow-up IVH(ml); Median (IQR)**	0 (0, 3.46)	0.94 (0.4, 3.90)	0.50 (0, 3.60)	0.310
**Follow-up total ICH volume (ml); Median (IQR)**	13.75 (6.90, 34.73)	8.21 (6.27, 13.19)	40.56 (14.70, 71.40)^c^	**0.0001**
**Follow-up PHE (5–23 HU, ml); Median (IQR)**	2.7 (1.2, 6.1)	1.5 (0.7, 2.8)	4.9 (2.5, 9.7)^c^	**0.008**
**Follow-up relative PHE (5–23 HU); Median ± IQR**	0.23 (0.11, 0.45)	0.29 (0.08, 0.49)	0.14 (0.10, 0.25)	0.101

^a^vs. HE *p* = 0.002

^b^vs. HE and HS *p* = 0.006

^c^vs. HS and HR *p*<0.022

### Perihematoma edema volume

**Fig 3:**
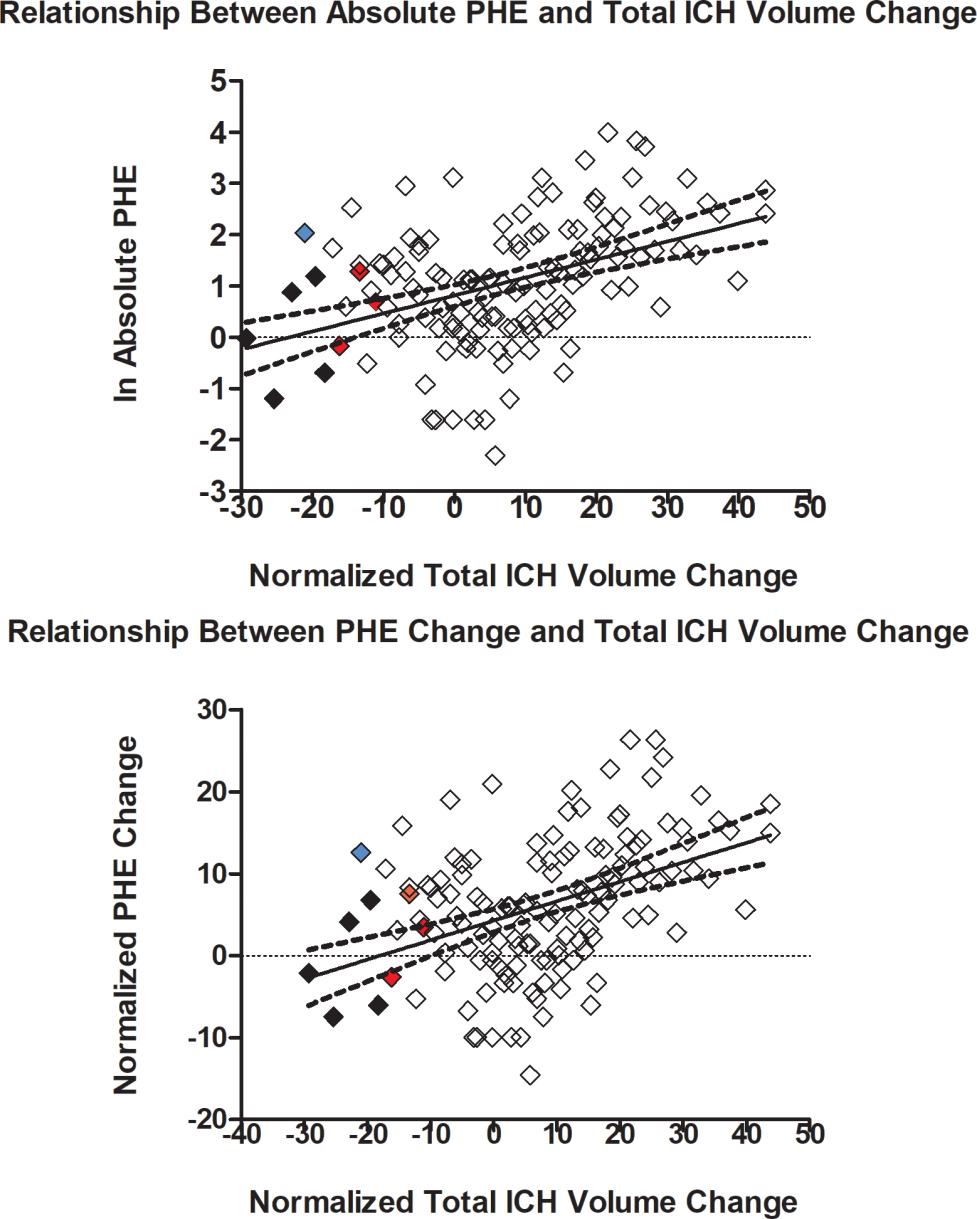
Top: Linear regression plot of normalized total ICH volume change and absolute PHE (R = 0.424, 95% CI [0.275, 0.553], *p* = 0.0001). Bottom: Linear regression plot of normalized total ICH volume change and normalized PHE change (R = 0.425, 95% CI [0.276, 0.554], *p* = 0.0001) The solid diamonds represent HR patients by absolute (blue), percent (red), and both (black) criteria.

Median (IQR) baseline PHE volume was 1.6 (0.8, 3.3) ml. Edema volume increased to 3.1 (1.3, 6.5) ml at follow up. Median PHE volume change in HR (defined as absolute volume decrease >3 ml) was 0.35 (-0.40, 51) ml and this was similar to HS patients (0.75 (0.10, 1.90); *p* = 0.501). Total ICH volume change, absolute PHE and PHE volume change were not normally distributed. After natural log transformation of absolute PHE volumes, and inverse-normalization of PHE volume change and total ICH volume change,[22] linear regression indicated that ICH volume change was predictive of follow-up absolute PHE volume and PHE volume change (Fig 3). HR was not associated with follow-up absolute PHE R = 0.294, 95% CI [-0.680, 0.892], *p* = 0.571) or PHE change R = 0.311, 95% CI [-0.669, 0.896], *p* = 0.548).

### Clinical outcomes

The rates of good clinical outcomes in HR and HS patients were similar when defined using both absolute (Table 2) and relative measures (Table 5). HR did not predict good outcome (OR = 0.864, [0.135, 5.542], *p* = 0.878). Good clinical outcomes were seen less often in patients with HE compared to other groups. The only predictors of good outcomes were follow-up total ICH volume (OR 0.864 [0.759 to 0.983], *p* = 0.027) and age (OR 0.954 [0.918 to 0.991], *p* = 0.015).

**Table 5:** Baseline clinical characteristics and outcomes (groups defined using relative volume change criteria).

	Stable Hematoma	Hematoma Retraction	Hematoma Expansion	P value
	HS, N = 90	HR, N = 8	HE, N = 38	
**Baseline**				
**Age; Mean ± SD (years)**	69 ± 13	67 ± 14	71 ± 15	**0.315**
**Male**	50 (55%)	6 (75%)	23 (61%)	**0.530**
**NIHSS, Median (IQR)**	10 (8, 16)	11 (10, 13)	14 (10, 18)^a^	**0.197**
**Baseline Systolic BP (mmHg); Median (IQR)**	178.3 (159, 199)	173.0 (148.5, 188.3)	183 (158, 200)	**0.600**
**Baseline Diastolic BP (mmHg); Median (IQR)**	90.50 (82, 106)	87.50 (80.75, 110)	88 (76.5, 110)	**0.801**
**Past History of Stroke**	19 (21.6%)	1 (12.5%)	5 (13.2%)	**0.483**
**Anticoagulant-associated ICH**	5 (6.1%)	2 (28.6%)	8 (22.9%)^a^	**0.015**
**Diabetes**	24 (27.6%)	2 (25%)	10 (26.3%)	**0.980**
**Glucose (mmol/l), Median (IQR)**	7.15 (5.75, 8.35)	6.65 (6.25, 7.75)	7.55 (5.85, 8.95)	**0.610**
**Hypertension**	67 (76.1%)	6 (75%)	28 (73.7%)	**0.958**
**Aspirin**	17 (21%)	2 (28.6%)	6 (18.2%)	**0.820**
**Outcome**				
**90 Day mRS**	3 (2, 5)	3 (3, 4)	5 (3, 6)^b^	**0.003**
**mRS 0–2**	30 (50.8%)	4 (66.7%)	7 (24.1%)^a^	**0.030**

^a^vs. HS *p*<0.025

^b^vs. HS and HR p<0.029

## Discussion

This is the first study to report early HR, which is a relatively rare phenomenon occurring in 4.4%–5.9% of patients within the first 24 hours. Furthermore, measurable HR appears to be more common in patients with IVH. Our results suggest that the apparent reduction in ICH volume is in most cases an artifact resulting from a decrease in the visible IVH component over time. The latter likely results from the flow of CSF within the ventricular system, which disperses blood. In contrast, the IPH components appear to be stable in most patients.

The physiological basis of HR may be successful hemostasis. Endothelial rupture and initiation of the coagulation cascade ultimately results in fibrinogen conversion to fibrin, forming a thrombus composed of platelets, red blood cells, and fibrin.[1,6,23–25] The platelet actin-myosin skeleton contracts due to integrins bound to fibrin, extruding plasma, resulting in HR.[26] In vitro, this process can be measured objectively; coagulation occurs within 2 hours, and is associated with a 40% reduction in clot volume.[26]

Extrusion of plasma from the retracting hematoma has been hypothesized to contribute to PHE formation.[7] Serial MRI measurements suggest that clot derived plasma is the major source of PHE formation in acute ICH.[27] The link between normal hemostasis and PHE is supported by lower PHE volumes in patients with thrombolysis related ICH.[28] Contrary to our hypothesis, we found no link between HR and PHE. This may reflect the acute nature of our study. It may be that more delayed serial CT measurements are required to demonstrate this effect, although the boundary between hematoma and PHE becomes less distinct over time, making this a challenge.

### Natural history of hematoma evolution

There are a limited number of studies of the natural history of late hematoma evolution in ICH patients. It is clear that HE occurs in up to 1/3 of patients, if the initial CT scan is obtained within 3 hours of onset, but after that hematoma volume remains stable for the first day in the majority of cases. In the subacute period, Serial CT studies indicate that the visible intraparenchymal hyperdensity begins to disappear within 5–14 days and becomes invisible 2–3 weeks after onset.[29]

### Perihematoma edema

A CT study performed in one human cadaver injected with 4 ml of blood in the basal ganglia indicated HR of approximately 45% and PHE volume increase of 100% HR and increase in PHE within 5 hours.[30] Several animal models also indicate HR and PHE development in the absence of active bleeding occurs within hours of ICH onset.[24,30] Conversely, we did not find HR within 24 hours or increased PHE in those patients that had a decrease in total hematoma volume.

In our study, HE of >6ml and >30% was seen in 29.4% and 27.8% of patients, respectively, which is similar to previously reported rates.[4,5] Expansion was associated with larger baseline ICH volumes, functional disability at 90 days, and anticoagulant use. Anticoagulant-associated ICH was more likely to be associated with HE. This has been described previously and is the rationale for rapid correction of the coagulopathy.[31–33] Interestingly, two HR patients had an AAICH. This is also consistent with the hypothesis that most apparent HR is due to IVH clearance. The presence of IVH has previously been described as confounder in the assessment of HE rates.[4]

This study does have limitations. There is currently no accepted definition of what constitutes HR, and that used in this study is somewhat arbitrary. This is an issue that has also affected studies of the rate of HE. We defined HR on the basis of absolute, rather than relative, volume changes, as, based on previous approaches to HE.[4] We chose values 50% of those used to define HE to define HR, based on the initial observation that retraction volumes are small and only noticed in the IVH and not the IPH component. Additionally, even though our ICC was 0.99, there is the possibility that the HR reports could have been affected by measurement error. Our exclusion criteria led to a small sample size in the HR group. Finally, a longer interval between onset and follow up imaging (e.g., 72 hours) may be required to demonstrate HR. We chose to assess early HR within 24 hours, as this time period has previously been demonstrated to be associated with stabilization of hematoma volume.[4,30]

## Conclusions

Early HR in ICH patients is rare and unrelated to prognosis. Hematoma retraction is not associated with PHE volume PHE and is unlikely to be a useful endpoint in clinical ICH studies.

## Supporting information

S1 File Raw data. (SAV)
